# Establishment and Characterization of a CTC Cell Line from Peripheral Blood of Breast Cancer Patient

**DOI:** 10.7150/jca.33157

**Published:** 2019-10-15

**Authors:** Pan Zhao, Wenbin Zhou, Chang Liu, Huirong Zhang, Zhiqiang Cheng, Weiqing Wu, Kaisheng Liu, Hong Hu, Caineng Zhong, Yayuan Zhang, Dongxian Zhou, Feiyuan Liu, Yong Dai, Jianhong Wang, Chang Zou

**Affiliations:** 1 Clinical Medical Research Center, the Second Clinical Medical College of Jinan University, the First Affiliated Hospital of Southern University, Shenzhen People's Hospital, Shenzhen 518020, China; 2 Department of breast and thyroid surgery, the Second Clinical Medical College of Jinan University, the First Affiliated Hospital of Southern University, Shenzhen People's Hospital, Shenzhen 518020, China; 3 Central Laboratory, Dalian Municipal Central Hospital, Dalian 116033, China; 4 Department of Pathology, the Second Clinical Medical College of Jinan University, the First Affiliated Hospital of Southern University, Shenzhen People's Hospital, Shenzhen 518020, China; 5 Shenzhen Public Service Platform on Tumor Precision Medicine and Molecular Diagnosis, the Second Clinical Medical College of Jinan University, Shenzhen People's Hospital, Shenzhen 518020, China; 6 Department of Health Management, the Second Clinical Medical College of Jinan University, the First Affiliated Hospital of Southern University, Shenzhen People's Hospital, Shenzhen 518020, China

**Keywords:** Circulating tumor cell, Breast cancer, Cancer stem cell, Primary culture

## Abstract

Background: Circulating tumor cell (CTC)-based patient-derived cells are ideal models for investigating the molecular basis of cancer. However, the rarity and heterogeneity of CTCs as well as the difficulties of primary culture limit their practical application. Establishing efficient *in vitro* culture methods and functionally characterizing CTCs is essential for cancer studies. To this end, we developed an experimental protocol for the isolation, expansion, and identification of breast cancer CTCs.

Methods: The CTC-3 cell line was established from peripheral blood cells of a breast cancer patient. A karyotype analysis was performed. The molecular profile was assessed by flow cytometry, quantitative real-time PCR, and western blot. The characteristics of tumors formed by CTC-3 cells were evaluated by cell growth and tumor sphere formation assays and in a mouse xenograft model. The tumors were analyzed by immunohistochemistry, immunofluorescence analysis, and hematoxylin and eosin staining.

Results: The CTC-3 cell line showed more aggressive growth both *in vitro* and *in vivo* than the widely used MCF-7 breast cancer cell line. CTC-3 cells were also more resistant to chemotherapeutic agents, and gene profiling indicated higher expression levels of the epithelial-to-mesenchymal transition and stemness markers as compared to MCF-7 cells.

Conclusions: CTC-3 cells are a better model for investigating the malignant behavior of breast cancer than existing cell lines.

## Introduction

Breast cancer is the most prevalent malignancy among women worldwide. Metastasis to bone, brain, liver, and lung accounts for the majority of breast cancer-related deaths 1. Tumor cell dissemination involves several steps starting with detachment from the primary tissue followed by vascular entry and travel to distant tissue where a secondary tumor can be initiated 2.

Established breast cancer cell lines are invaluable tools for identifying and characterizing the molecules and pathways that control tumor growth and metastasis 3. Since the establishment of the first human breast carcinoma cell line in 1958 4, the most widely used breast cancer cell lines were originally from Caucasians or African Americans including T47D, MDA-MB-231, and MCF-7 3. However, since tumor cells are typically isolated from a single site in the tumor by biopsy, the resultant cell line does not exhibit the heterogeneity of the primary tumor. Moreover, tumor cells do not reflect the molecular changes that occur during cancer progression. The translational relevance of cell lines is also questionable because prolonged culture and multiple passaging can induce epigenetic and molecular phenotypes that are not representative of the original tumor 5.

Circulating tumor cells (CTCs) are rare tumor cells that leave the primary tumor and circulate into the blood of cancer patients 5, 6. Detection of CTCs may be useful for early breast cancer diagnosis since it bypasses the need for ionizing radiation (mammography) or invasive biopsy 7, 8. CTCs could also embody the molecular heterogeneity of a primary cancer and their metastasis is more tolerable to patients 9, 10. The potential of CTCs as minimally invasive biomarkers for predicting patient prognosis and monitoring recurrence and as targets for precision therapeutics has been demonstrated in several studies 11-14.

Several breast cancer CTC cell lines have been generated from cancer patients and used to monitor response to anti-cancer drugs 15. Drug sensitivity measurements were concordant with clinical history including sensitivity to paclitaxel and capecitabine and resistance to fulvestrant, doxorubicin, and olaparib 16. CTCs were used to develop a drug sensitivity test and screening system that could guide drug discovery for personalized treatment 17. However, the practical application of CTCs is hindered by their rarity and heterogeneity and the difficulties of primary culture. Developing more efficient *in vitro* culture methods is essential for establishing CTC cell lines that recapitulate the characteristics and behavior of the original tumor.

In this study we describe the establishment of a CTC cell line derived from naturally transformed breast cancer cells obtained from a 42-year-old Chinese woman diagnosed with breast carcinoma. Our cell enrichment technique is based on the removal of red blood cells by chemical lysis and the magnetic depletion of normal hematopoietic cells labeled with an anti-CD45 antibody/magnetic nanoparticle complex. The novel CTC-3 cell line was characterized in terms of biological and molecular features and karyotype, and tumorigenic potential was evaluated *in vitro* and in mice. We also analyzed the response of CTC-3 cells to different first-line drugs for the treatment of breast cancer.

## Materials and Methods

### Patient samples and blood collection

After obtaining informed consent, peripheral blood was collected from patients with advanced metastatic breast cancer. Blood was collected in EDTA tubes (10 ml) and was used for *in vitro* CTC culture (Supplementary Materials and Methods).

### Cell culture

MCF-7, T47D, and MDA-MB-231 breast cancer cell lines were cultured as described in the Supplementary Materials and Methods.

### Immunofluorescence analysis

Immunofluorescence labeling was performed using fluorescein isothiocyanate (FITC)-conjugated anti-pan cytokeratin (CK) (ab215838) and phycoerythrin (PE)-conjugated anti-cluster of differentiation (CD)45 (ab10558) antibodies and Fluoroshield Mounting medium with 4',6-diamidino-2-phenylindole (DAPI; ab104139) (all from Abcam, Cambridge, MA, USA). Cells were fixed and permeabilized by incubation for 20 min in 4% paraformaldehyde and 0.2% Triton X-100, respectively. A mixture of 10 μg/ml anti-CD45 and 10 μg/ml anti-CK antibodies and 500 nM DAPI were added to a microfluidic device followed by incubation for 20 min. After washing, the device was examined and only cells that were positive for DAPI and CK and negative for CD45 (DAPI+/CK+/CD45-) with appropriate size and morphology were counted as CTCs 17.

### Karyotyping

The karyotyping protocol is described in the Supplementary Materials and Methods.

### Subcutaneous tumorigenicity assay

To compare the tumorigenicity of CTC cells to that of MCF-7 cells, female immunodeficient mice (8 weeks old, n = 12; Medical Laboratory Animal Center, Guangdong Province) were divided into two groups that were subcutaneously injected in the left and right shoulders with 10^6^ CTC-3 and MCF-7 cells, respectively, resuspended in 100 µl medium. Tumor growth was monitored and tumor volume (mm^3^) was measured weekly using electronic calipers and calculated with the formula (length × width × height)/2. Tumor growth (mean ± SD of three independent animals) was plotted as a function of time. Animal experiments were performed in accordance with the guidelines for laboratory animal use and were approved by the Animal Experimentations Ethnics Committee.

### Cell growth analysis

CTC-3 and MCF-7 cells were cultured in complete growth medium. When they reached 70%-80% confluence, the cells were trypsinized and resuspended at a density of 5.5 × 10^3^ cells/ml; a 1-ml cell suspension was added to each well of a 24-well plate. The cells were trypsinized and counted on days 3, 5, and 7 of culture (n = 3).

### Tumor sphere formation assay

CTC-3 and MCF-7 cells were cultured in complete growth medium. When they reached 70%-80% confluence, the cells were trypsinized and resuspended in cancer stem cell (CSC) medium (Gibco, Grand Island, NY, USA) consisting of Dulbecco's Modified Eagle's Medium (DMEM)/F12 supplemented with epidermal growth factor, basic fibroblast growth factor, insulin, B27, and knock-out serum. The cells were seeded at a density of 1 × 10^4^ cells/well in ultra-low attachment 6-well plates. After 14 days of culture under normoxic conditions with replenishment of the medium every 3 days, tumor spheres were formed.

### Immunocytochemistry

Paraffin-embedded tumor tissue samples including primary tumor and lymph node biopsies from breast cancer patients and subcutaneous CTC-3 cell xenografts in immunodeficient mice were cut into 3 mm-thick sections and analyzed for estrogen receptor (ER), progesterone receptor (PR), erbB-2, E-cadherin and Ki-67 expression using appropriate antibodies (Supplementary Materials and Methods).

### Western blot analysis

CTC-3, T47D, MBA-MD-231, and MCF-7 cells were lysed in lysis buffer containing protease and phosphatase inhibitors (Keygentec, Nanjing, China). Protein concentrations were quantified by BCA kit (Keygentec). Equal amount of protein was loaded in each lane. Constant voltage electrophoresis was carried out with 10% polyacrylamide gels and then transferred to polyvinylidene fluoride (PVDF) membranes (Merk, Massachusetts, USA). POVDF membranes were blocked with 3% BSA (Sigma) and hybridized with Estrogen Receptor (1:1000, Abcam, Cambridge, UK), Progesterone Receptor (1:1000, Abcam), Her-2 (1:1000, Abcam), or β-actin (1:2000, Abcam) primary antibodies overnight at 4 ˚C. After washed with TBST (Keygentec), the PVDF membranes were incubated with secondary antibodies (ZSGB-bio, Beijing, China) at room temperature for 60 minutes, followed by washing with TBST. PVDF membranes were covered with 3,3-diaminobenzidine (DAB) for the display of specific protein bands.

### Drug resistance test

CTC-3 cells seeded at an initial density 1 × 10^4^ cells/well were maintained in complete medium at 37°C in a humidified atmosphere of 5% CO_2_. After 12 h, the cells were treated with cyclophosphamide, paclitaxel, or pirarubicin for 24, 48, and 72 h at concentrations of 0.01, 0.1, 1, 10, and 100 μg/ml. The viability of surviving cells was evaluated with the Cell Counting Kit 8 assay 18.

### Flow cytometry

Flow cytometry was performed with PE-conjugated mouse anti-human CD44 and FITC-conjugated mouse anti-human CD24 antibodies (both from BD Biosciences, San Diego, CA, USA) on a FACS Canto II instrument (BD Biosciences, San Diego, CA, USA), and data were analyzed with Cell Quest software (BD Biosciences).

### RNA-sequencing (RNA-seq)

Total RNA was isolated from cells using TRIzol reagent (Invitrogen, Carlsbad, CA, USA) and purified using the NanoPhotometer spectrophotometer (Implen, Westlake Village, CA, USA); RNA concentration and integrity were estimated using the Qubit 2.0 flurometer (Life Technologies, Carlsbad, CA, USA) and Agilent 2100 Bioanalyzer (Agilent Technologies, San Diego, CA, USA), respectively. Polyadenylated RNAs were isolated using Next Magnetic Oligo dT 25 beads (New England Biolabs, Ipswich, MA, USA), and first-strand synthesis was performed using the Next RNA First Strand Synthesis Module (New England Biolabs). Directional second strand synthesis was performed using Next Ultra Directional Second Strand Synthesis Module (New England Biolabs). The Next DNA Library Prep Master Mix Set for Illumina (New England Biolabs) was used to prepare expression libraries according to the manufacturer's protocol. Accurate quantification for sequencing applications was performed using the quantitative real-time qRT-PCR-based KAPA Biosystems Library Quantification Kit (Kapa Biosystems, Wilmington, MA, USA). Paired-end sequencing (75 bp) was performed on a NextSeq 500 sequencer (Illumina, San Diego, CA, USA). RNA-seq reads were aligned to the human genome (hg19) using TopHat2 16, and the number of reads mapped to each gene was calculated with HTseq (http://htseq.readthedocs.io/). Differentially expressed genes between CC and CC-CR were detected with edgeR based on a negative binomial distribution. P values were adjusted with Benjamini and Hochberg's multiple test correction procedure. Differential expression was determined based on |log2 (fold-change)| > 1 and false discovery rate < 0.01. Data analysis is described in Supplementary Materials and Methods.

### qRT-PCR analysis

Total RNA was isolated from cells using an RNA purification kit (Corning Inc., Corning, New York, USA) according to the manufacturer's instructions. Reverse transcription was performed using the PrimeScript RT Reagent Kit (TaKaRa, Shiga, Japan). qRT-PCR was carried out with SYBR Premix Ex Taq (Perfect Real Time) (Takara Bio) on a LightCycler 96 System (Roche, Basel, Switzerland). The primers used in this study are listed in Doc S1. GAPDH was used as an internal control. The results are presented as the expression ratio of target sample to control sample for each group, which was calculated with the cycle threshold method (2^-∆∆CT^).

### Cell invasion assay

The invasiveness of CTC-3 cells was evaluated with the Matri-gel invasion assay as previously described 19 using transwell inserts (8 μm pore size) (BD Biosciences) in a 24 well plate. CTC-3 and MCF-7 cells (10^5^ cells/insert) were seeded on top of the Matri-gel in serum-free minimal essential medium (MEM) and DMEM/F12. The lower chamber was filled with MEM/10% fetal bovine serum (FBS) and DMEM/F12/10% FBS. After incubation at 37°C for 48 h, CTC-3 and MCF-7 cells that had migrated through the membrane were stained with crystal violet and counted under a microscope.

### Statistical analysis

Data are expressed as the mean ± SD of three independent experiments. Statistical analysis was performed using Prism v.7.0 (GraphPad, La Jolla, CA, USA), Origin 8 (OriginLab, Northampton, MA, USA), and Excel (Microsoft, Redmond, WA, USA) software. Results are expressed as mean ± SD of at least three independent experiments. The significance of differences among groups was assessed with the two-tailed Student's t test for normally distributed data, and P < 0.05 and < 0.01 were considered significant and highly significant, respectively.

## Results

### *In vitro* culture of CTCs isolated from metastatic breast cancer patients

**Figure 1:**
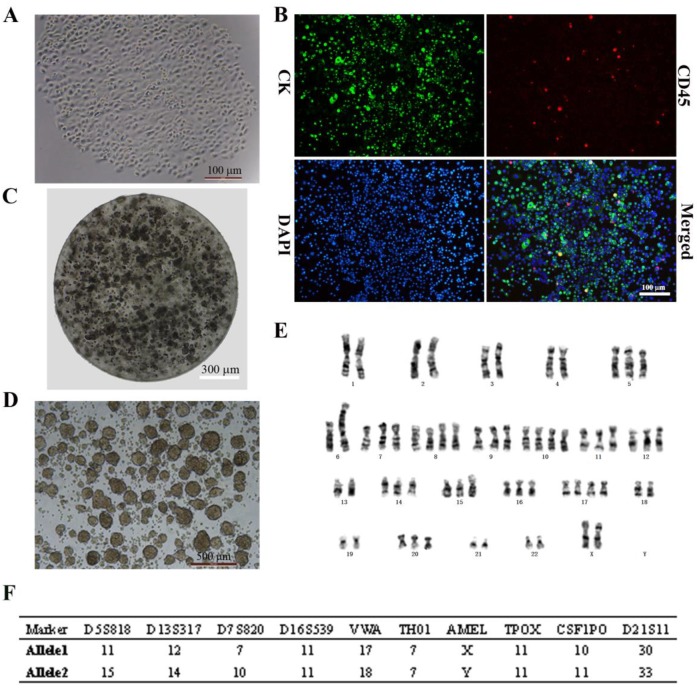
*In vitro* expansion of breast cancer CTCs. **(A)** Morphology of the first generation of CTC-3 cells which grown as a single clone. **(B)** Immunocytochemical detection of DAPI+/CK+/CD45- CTC spheres. **(C)** Morphology of CTC-3 cell spheres embedded in alginate hydrogel. **(D)** *In vitro* tumor sphere formation assay of CTC-3 cells. **(E)** Karyotyping of CTC-3 cell line. **(F)** STR analysis of CTC-3 cells and PBMCs from the same patient.

CTCs began to proliferate and form clusters after 14 days of culture (Fig. 1A). Immunocytochemical analysis revealed DAPI+ cells that expressed the epithelial marker CK and were negative for the leukocyte marker CD45 (DAPI+/CK+/CD45-; > 95% of cells), indicating that CTCs were of epithelial origin according to the standard definition (Fig. 1B). This stable breast cancer CTC line was named CTC-3, and has been growing in culture for 2 years with a short doubling time. CTC-3 cells formed tumor spheres when encapsulated in alginate gel beads (Fig. 1C). Besides when cultured in an ultra-low attachment 6-well plate in CSC medium, the cells formed tumor spheroids with a mean diameter of 200 μm (Fig. 1D). Karyotyping revealed that the chromosome number of CTC-3 varied from 56 to 60 and that the cells were aneuploid (Fig. 1E). Moreover, short tandem repeat (STR) analysis indicated that CTC-3 cells and peripheral blood mononuclear cells (PBMCs) from the donor showed > 83% similarity (Fig. 1F). The CTC-3 cell line can be frozen, banked, and thawed and successfully regrown.

### Comparison of CTC-3 cells with patient tumor and xenograft tissues

**Figure 2:**
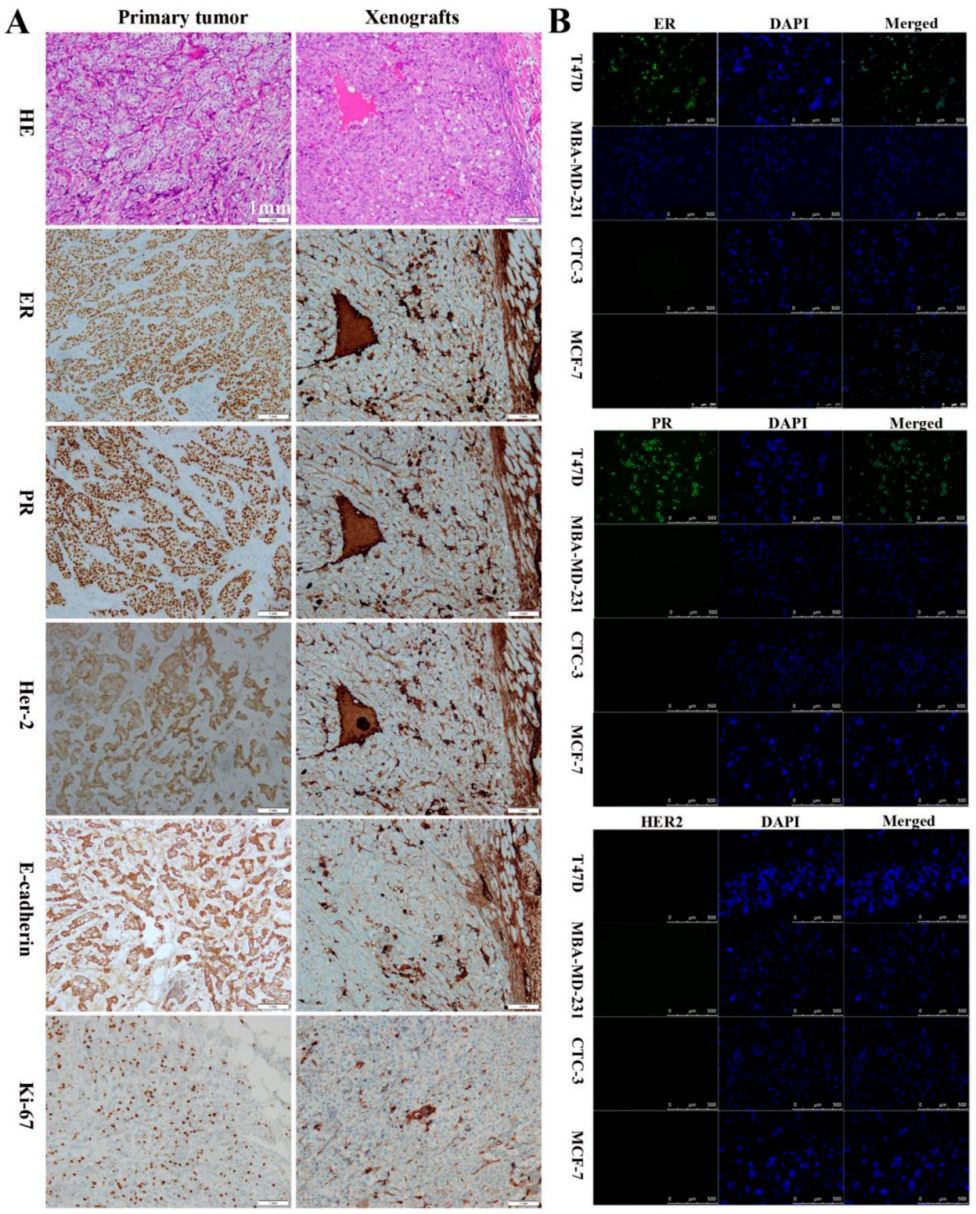
Comparison of the CTC-3 cells with patient tumor tissues and mouse xenografts. **(A)** Hematoxylin and eosin staining and immunocytochemical detection of ER, PR, Her-2, E-cadherin, and Ki-67 in primary tumor tissues and xenografts. **(B)** Immunofluorescence analysis of ER, PR, and Her-2 expression in the TD47, MDA-MB-231, MCF-7, and CTC-3 cells.

To assess their tumorigenic potential, CTC-3 and MCF-7 cells were subcutaneously injected into immunodeficient mice. Her-2, ER, and PR expression in the xenografts as well as in the primary tumor and lymph node of breast cancer patients was examined. In primary tumors, the rates of positivity were 90% for ER, 80% for PR, and 2+ for Her-2 (Figs. 2A), which was similar with that of the lymph node metastasis (Figure S1A). In contrast, xenografts from mice were negative for ER and PR and positive for Her-2 expression (Fig. 2A). All tissues presented the features of differentiated adenocarcinoma cells, including low nucleus-to-cytoplasm ratio, irregular nuclear shape, and vacuoles. E-cadherin and Ki-67 were detected in primary tumors and xenografts (Fig. 2A), while lymph nodes and xenografts expressed CK (Fig. S1B). Additionally, xenografts were positive for carcinoembryonic antigen (CEA) and negative for p63 (Fig. S1C). Immunofluorescence analysis revealed that CTC-3 cells were negative for ER, PR, and Her-2 (Fig. 2B), which is similar to the negative control MDA-MB-231 cells. On the other hand, the positive control T47D cells expressed both ER and PR.

### Growth characteristics of CTC-3 cells

**Figure 3:**
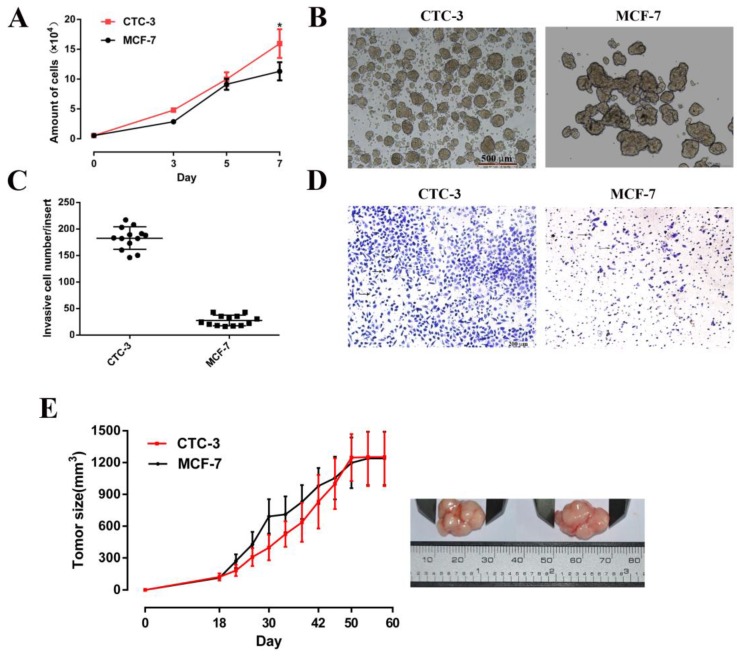
Proliferation and tumor sphere formation capacity of CTC-3 and MCF-7 cells. **(A)** Growth curve of CTC-3 and MCF-7 cells. Cells were counted every 2 days from three randomly selected replicates. **(B)** Gross morphology of tumors formed by CTC-3 and MCF-7 cells *in vivo* and change in tumor volume change over 60 days. **(C)** Matri-gel invasion assay of CTC-3 cells; the total number of migrated CTC-3 cells per transwell insert is shown. *P < 0.001 vs. control MCF-7 cells. **(D)** CTC-3 cells that have migrated through the transwell insert. Black arrows indicate cells stained with crystal violet. **(E)** Tumor sphere formation assay for CTC-3 and MCF-7 cells.

The growth rate of CTC-3 and MCF-7 cells was compared *in vitro* and *in vivo*. Both cell lines grew rapidly, with cell numbers that were 29 and 20.56 times higher, respectively, than the initial values after 7 days of culture. However, the number of CTC-3 cells was higher than that of MCF-7 cells on day 7 (Fig. 3A). In addition, compared with the MCF-7, the number of CTC-3 cells migrated through Matri-gel-coated membranes was increased by 6.53-fold (Fig. 3C, D). The results of the xenograft assay showed that the tumorigenic potential of CTC-3 and MCF-7 cells was comparable (Fig. 3E), although CTC-3 cells formed a greater number of tumor spheres that were more compacted when cultured in CSC medium (Fig. 3B).

### Drug sensitivity of CTC-3 cells

**Figure 4:**
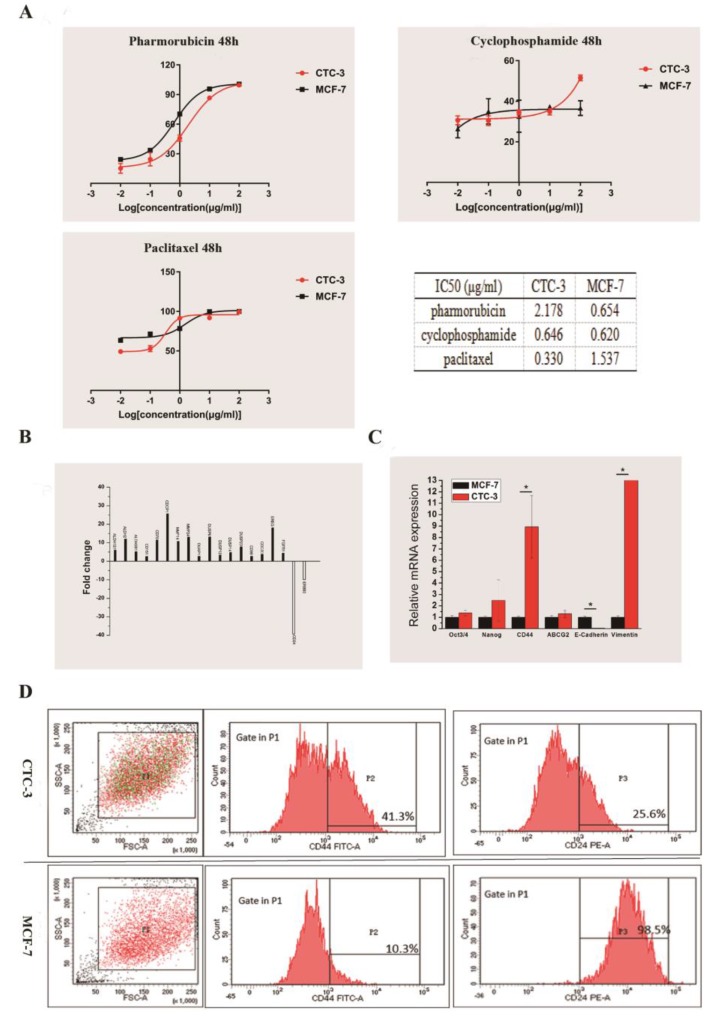
Drug sensitivity and gene expression profile of CTC-3 cells. **(A)** Drug sensitivity and IC_50_ of CTC-3 and MCF-7 cells treated with indicated concentrations of cyclophosphamide, paclitaxel, and pirarubicin for 48 h. **(B)** Gene expression profiles of CTC-3 and MCF-7 cells by RNA-seq. **(C)** qRT-PCR analysis of stemness markers octamer-binding transcription factor Oct 3/4, Nanog, CD44; drug resistance marker ABCG2; and EMT-related markers E-cadherin and Vimentin in CTC-3 and MCF-7 cells. *P < 0.05 vs. MCF-7 cells. Error bars represent SD of three replicates of three separate samples (n = 9). (D) Flow cytometry analysis of CD44 and CD24 expression in CTC-3 and MCF-7 cells.

We compared the drug sensitivity of CTC-3 and MCF-7 cells to the first-line anti-breast cancer drugs pirarubicin, paclitaxel, and cyclophosphamide. The rate of growth inhibition rate was higher for MCF-7 than for CTC-3 cells treated with at 0.01, 0.1, 1, 10 μg/ml pirarubicin for 48 h, with significant differences observed at concentrations of 1 and 10 μg/ml (70.22% ± 1.52% vs. 45.83% ± 3.12% and 95.63% ± 1.70 % vs. 86.59% ± 0.53%, respectively) (Fig. 4A). However, at 100 μg/ml pirarubicin, the inhibition rate was 100% for both cell lines. The calculated the half-maximal inhibitory concentration (IC_50_) of CTC-3 and MCF-7 was 2.178 and 0.654 μg/ml, respectively (Fig. 4A).

The inhibition rates of MCF-7 cells treated with 0.01 and 0.1 μg/ml paclitaxel (63.38% ± 2.57% and 71.45% ± 1.86%, respectively) were higher than those of CTC-3 (49.24% ±1.41% and 52.89% ± 3.76%, respectively) (Fig. 4A). However, at 1 μg/ml, the rate was higher for CTC-3 than for MCF-7 cells (91.43% ± 0.57% vs. 78.37% ± 2.06%) (Fig. 4A). There were no significant differences between the two cell lines at paclitaxel concentrations of 1 and 10 μg/ml. The IC_50_ values of CTC-3 and MCF-7 were 0.3304 and 1.537 μg/ml, respectively (Fig. 4A).

The inhibition rate of MCF-7 and CTC-3 cells treated with cyclophosphamide did not differ significantly at concentrations of 0.01, 0.1, 1, and 10 μg/ml (Fig. 4A). However, at 100 μg/ml cyclophosphamide, the inhibition rate was higher for CTC-3 than for MCF-7 (51.51% ± 1.43% vs. 36.60% ± 3.61%) (Fig. 4A). The calculated IC_50_ values of CTC-3 and MCF-7 cells were 0.646 and 0.620 μg/ml, respectively (Fig. 4A). These results indicate that CTC-3 cells are more resistant to pirarubicin and cyclophosphamide but more sensitive to paclitaxel than MCF-7 cells.

### Gene expression profile of CTC-3 cells

We performed RNA-seq of CTC-3 and MCF-7 cells to evaluate global changes in the expression of genes related to CSCs, drug resistance, and metastasis. We found that 17 genes were upregulated and two were downregulated in CTC-3 as compared to MCF-7 cells (Fig. 4B). In addition, the qRT-PCR results showed that the levels of CSC markers Oct3/4, Nanog, and especially CD44 were higher in CTC-3 cells than in MCF-7 (Fig. 4C). ATP-binding cassette super-family G member (ABCG)2 is closely related to drug resistance of tumor cells; here we found that ABCG2 was more highly expressed in CTC-3 than in MCF-7 cells, although the difference was non-significant. Interestingly, the levels of the epithelial-to-mesenchymal transition (EMT)-related markers E-cadherin was significantly lower and Vimentin was significantly higher, respectively, in CTC-3 as compared to MCF-7 cells, indicating that the former undergo EMT and thus have higher metastatic potential.

### CSC-related marker expression in CTC-3 cells

Cells with high CD44 and low CD24 expression (CD44+/CD24-) define a CSC-like population in breast tumors 20 that exhibits high tumorigenic potential and drug resistance. The ratio of CSC-like cells in CTC-3 and MCF-7 cell lines was compared by flow cytometry analysis of CD44+ and CD24- cell ratio. In the CTC-3 cell line, 42.9% ± 1.98% of cells were CD44+ and 78.85% ± 15.34% were CD24- (Fig. 4D). In contrast, only 21.15% ± 15.34% MCF-7 cells were CD44+ but nearly all were CD24+ (99.15% ± 0.92%) (Fig. 4D). These results indicate that the CSC population is larger in CTC-3 than in MCF-7 cells.

## Discussion

CTCs are seed cells disseminated from the original tumor via the circulation that are responsible for metastasis and relapse in cancer 21-23. Detection of CTCs and/or CTC clusters by immunolabeling or karyotyping has provided insight into the clinical significance of CTCs 24-26. However, their correlation with tumor progression and patient prognosis remains unclear 27, 28. CTCs are rare in blood and have polytrophic cell morphology and multiple cellular origins 29, 30. For these reasons, CTCs are the ideal cell model for investigating the heterogeneity, metastasis, and stemness of solid tumors 31,32. To this end, establishing CTC-based cell cultures and stable cell lines is critical for elucidating the functions of CTCs. In the present study, peripheral blood samples from 50 patients (16 breast cancer patients, 18 stomach cancer, 16 bowel cancer) were cultured (Doc S1), and we established the novel CTC-3 breast cancer cell from cells isolated from the peripheral blood of a breast cancer patient and characterized its features including tumorigenic potential *in vitro* and *in vivo*.

White blood cells and thrombocytes may promote the survival of CTCs 33. To avoid loss of rare CTCs during separation, we seeded all PBMCs in a Matri-gel-coated 6-well plate after removing red blood cells from 6 ml of whole peripheral blood. Although debris from non-cancer cells could inhibit CTC growth, our results demonstrate that mimicking the *in vivo* tumor microenvironment as closely as possible may be important for successfully establishing CTC cultures.

Efforts to identify CTCs and their cellular origin have been hampered by the heterogeneity of solid tumors; to this end, descriptive studies of cell morphology, protein expression, karyotype, and STR identification are necessary. Functional analyses are equally important for characterizing CTCs; in this study, we carried out *in vitro* cell quantification and evaluation of anchorage-independent growth, spheroid formation, *in vivo* tumorigenicity, and drug resistance. Even after more than 13 passages, CTC-3 continued to be CK+/CD45- with a karyotype and STR profile that was similar to those of the patient's white blood cells. Moreover, hematoxylin and eosin staining revealed that CTC-3 cell xenografts showed pathological features that were similar to those of the patient tumor tissue. These results confirm that CTC-3 is a PDC line originating from breast cancer CTCs. The cell growth assay showed that CTC-3 has a more aggressive and CSC-like phenotype than MCF-7 cells, which are also derived from metastasis breast cancer tissue. Gene profiling by RNA-seq and qRT-PCR indicated that genes related to cell invasion and stemness were upregulated in the CTC-3 cell line relative to MCF-7 cells. Thus, CTC-3 cells may be a better cell model for studying breast cancer metastasis and stemness. In addition, the *in vitro* cell viability assay showed that CTC-3 cells are more resistant to first-line chemotherapies than MCF-7 cells, which was consistent with the observed upregulation of the multidrug-resistance gene ABCG2. Notably, the pathological diagnosis of the donor patient was ER+/PR+ and human epidermal growth factor receptor 2 (HER2) negative, and peripheral blood for CTC isolation was collected before any chemotherapy. On the other hand, western blot and flow cytometry analyses showed that the connective passages of CTC-3 cells were all triple-negative breast cancer cells, which was confirmed in CTC-3-derived xenografts by immunohistochemistry. These results underscore the heterogeneity of solid tumors and can potentially explain the failure of selective estrogen receptor modulator (SERM) treatment in ER-positive breast cancer patients. Following SERM treatment, the donor patient has achieved stable disease. However, based on our findings, in the case of relapse, first-line drugs may not be the best choice for this individual. We are currently attempting to isolate additional CTCs from this patient; once cultures are established, more in-depth analyses may be possible that could elucidate the molecular basis for relapse and metastasis.

## Supplementary Material

Supplementary figures and tables.
