# Supplementary material for: Establishment and Characterization of a CTC Cell Line from Peripheral Blood of Breast Cancer Patient
Source: J Cancer. 2019 Oct 15;10(24):6095–104. doi: 10.7150/jca.33157 (PMC6856591; doi:10.7150/jca.33157)
Supplement: Supplementary file 1 — Supplementary figures and tables. [file jcav10p6095s1.pdf]

Supplementary table

Table S1 Primer pairs used for quantitative real-time PCR studies

**Gene**	**Sequence(5'-3')**	**Gene ID**	**Product length (bp)**
β-actin	TGGCACCCAGCACAATGAA<br>CTAAGTCATAGTCCGCCTAGAAGCA	NM_001101.3	187
Oct3/4	GACAGGGGGAGGGGAGGAGCTAGG<br>CTTCCCTCCAACCAGTTGCCCCAAAC	NM_002701.4	119
Nanog	TCCAACATCCTGAACCTCAGCTA<br>AGTCGGGTTCACCAGGCATC	NM_024865.2	186
CD44	GACGAAGACAGTCCCTGGAT<br>CTTCTTGACTCCCATGTGAG	NM_000610.3	139
ABCG2	GCAAGCATCTATCCAGGTCAGG<br>GAAACACAACACTTGGCTGTAGCA	NM_001257386.1	173
E-cadherin	GAGTGCCAACTTGGACCATTCAGTA<br>AGTCACCCACCTCTAAGGCCATC	NM_004360.3	86
Vimentin	GGTGGACCAGCTAACCAACGA<br>TCAAGGTCAAGACGTGCCAGA	NM_003380.3	183

Table S2 the patient characteristics of clinical diagnosis

N=50	Patients	Age	Gender	Pathological TNM stage
1	Breast cancer	41	Female	T1N0M0 1A
2	Breast cancer	50	Female	PT1N0M0 1A
3	Breast cancer	24	Female	PT2N1M0 2B
4	Breast cancer	40	Female	PT1N2M0 3A
5	Breast cancer	44	Female	T2N2M0 3A
6	Breast cancer	30	Female	PT2N3M0 3B
7	Breast cancer	56	Female	PT1aN0M0 1A
8	Breast cancer	51	Female	PT1N0M0 1A
9	Breast cancer	52	Female	PT2N1M0 2B
10	Breast cancer	37	Female	PT2N2M0 3A
11	Breast cancer	47	Female	PT1N0M0 1A
12	Breast cancer	44	Female	PT1N0M0 3C
13	Breast cancer	67	Female	PT2N0M0 2A
14	Breast cancer	48	Female	T2N1M1
15	Breast cancer	54	Female	PT2N1M0 2B
16	Breast cancer	42	Female	PT1N0M0 2B
17	Stomach cancer	47	Male	T3N1M0
18	Stomach cancer	53	Male	T3N1M0
19	Stomach cancer	43	Female	T1N0M0
20	Stomach cancer	70	Female	T3N3M0
21	Stomach cancer	67	Male	T3N3M0
22	Stomach cancer	60	Female	T1N0M0
23	Stomach cancer	67	Male	T4N2M0
24	Stomach cancer	65	Male	T4N2M0
25	Stomach cancer	67	Male	T4N2M1
26	Stomach cancer	65	Male	T3N1M0
27	Stomach cancer	72	Female	T3N1M1
28	Stomach cancer	40	Male	T1N0M0
29	Stomach cancer	63	Male	T2N0M0
30	Stomach cancer	58	Female	T1N0M0
31	Stomach cancer	63	Female	T4N2M0
32	Stomach cancer	75	Female	T3N1M0
33	Stomach cancer	36	Female	T1N0M0
34	Stomach cancer	32	Female	T4aN0M0
35	Bowel cancer	68	Male	T3N1M0
36	Bowel cancer	44	Male	T3N1M0
37	Bowel cancer	51	Male	T2N0M0
38	Bowel cancer	76	Female	T3N0M0
39	Bowel cancer	51	Female	T2N0M0
40	Bowel cancer	63	Male	T2N0M0
41	Bowel cancer	52	Male	T4N2M0
42	Bowel cancer	50	Female	T3N1M0

43	Bowel cancer	60	Male	T4N3M0
44	Bowel cancer	33	Male	T3N1M0
45	Bowel cancer	55	Male	T3N1M0
46	Bowel cancer	60	Male	T3N0M0
47	Bowel cancer	76	Female	T3N0M0
48	Bowel cancer	61	Female	T3N0M0
49	Bowel cancer	60	Male	T2N0M0
50	Bowel cancer	64	Female	T2N1M0

Supplementary figure

Figure S

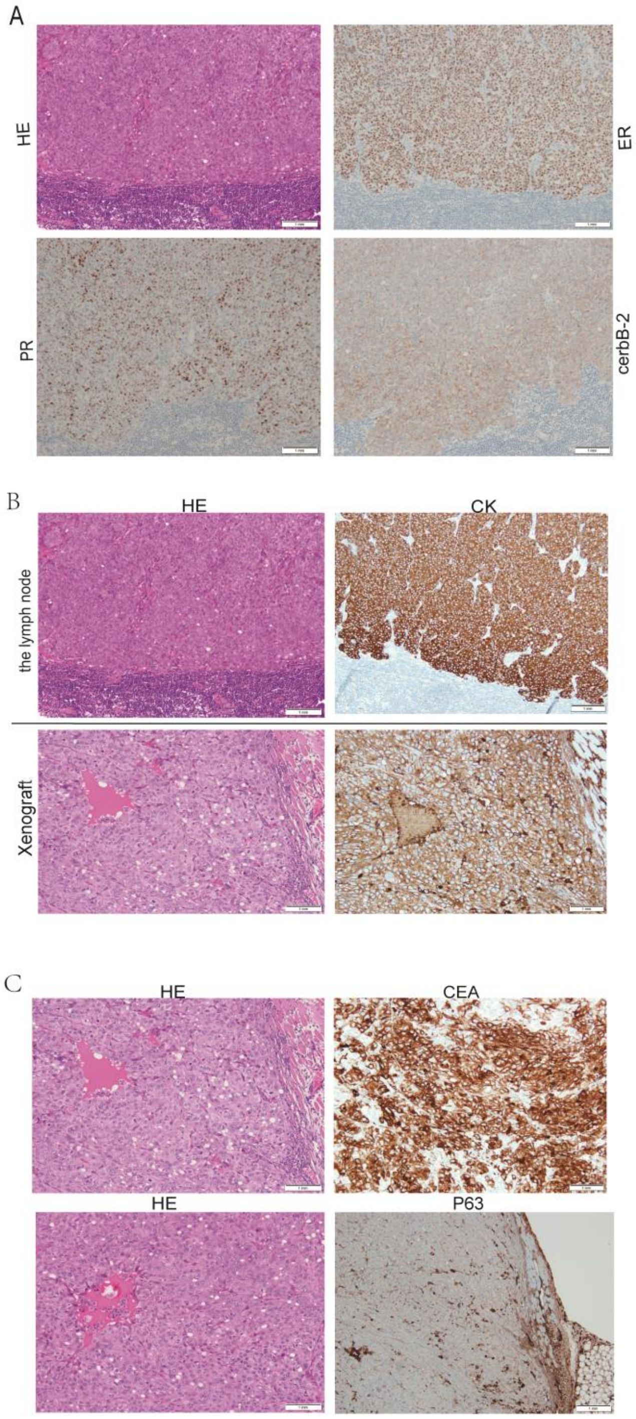

Figure S. H&E staining and immunohistochemical analysis of metastatic lymph node and xenograft. (A) ER, PR and cerbB-2 expression in metastatic lymph node. (B) Pan-CK expression in the metastatic lymph node and xenograft. (C) CEA and p63 expression in xenograft.

Supplementary Materials and methods

Ex vivo CTC culture

Peripheral blood (6mL) was obtained from the advanced breast cancer patient without chemotherapy and the blood was placed in an EDTA tube (BD). Ficoll-Hypaque gradient centrifugation was used to isolate the peripheral blood mononuclear cells (PBMCs) from the blood. The PBMCs after disposed the red cells were cultured in Matrigel-coated 6-well plate with the CCC medium (DMEM:1640=1:1, 10% FBS, 10% Nu-serum, 2mM L-Glutamine, 20 ng/mL EGF, 20 ng/mL FGF), and the cells were then cultured at 37 ℃, 5% CO_2 with changing the medium every 2-3 days. After about 14 days in CCC medium, cell clones appeared then were transferred into 6-well plates for further growth and then into T25 flasks for culture expansion. CTCs under these conditions could be quickly expanded and after a few months we obtained billions of tumor cells and established a colon CTC line.

Cell culture

MCF-7 and MDA-MB-231 cell lines were maintained in Dulbecco's Modified Eagle's Medium (DMEM, Gibco) supplemented with 10% fetal bovine serum (FBS) (Thermo Scientific, Pittsburgh, PA, USA). T47D were maintained in RPMI-1640 medium (Gibco) supplemented with insulin (10ug/mL) and 10% fetal bovine serum (FBS). All cultures were maintained at 37 ℃ in a humidified atmosphere of 5% CO_2 in air. Cells were harvested at approximately 90% confluence for further use.

Karyotyping assay

80 μL (20 μg/mL) colcemid (Gibco, 152120-12) was added to the medium of

CTC-3. Cells were harvested with trypsin after 3 h, incubated in hypotonic 75 mM KCl solution for 5 min, fixed in a mixture of methanol and acetic acid (3:1) overnight, spread on slides, slides were baked at 70 ℃ overnight, G-banding using trypsin and Giemsa, analyzed by GSL-120 automatic imaging system, and at least 10 metaphase cells were analyzed by the CytoVision Version 7.5 software.

Immunocytochemistry staining

Paraffin-embedded tumor tissues: (i) primary tumor biopsy of the breast cancer patient, (ii) lymph node biopsy of the breast cancer patient and (iii) subcutaneous CTC-3 xenografts in immunodeficient mice were cut in 3 μm sections. Human xenograft tumors were identified with the Anti-Estrogen Receptor alpha(1:1000, Abcam, Cambridge, UK), Anti-ErbB 2 antibody (1:500, Abcam, Cambridge, UK), Anti-Progesterone Receptor antibody (1:100, Abcam, Cambridge, UK), anti-Ki67 antibody (1:150, Abcam, Cambridge, UK), anti-Ecadherin antibody (1:100, Abcam, Cambridge, UK). For light microscopy, tissue sections were first deparaffinized and rehydrated in xylene and serial alcohol solutions, respectively, and then stained with Hematoxylin-Eosin (H&E) using the Dako Autostainer. For immunohistochemical staining, paraffin-embedded tissue sections were treated with the BenchMark ULTRA (Ventana Medical Systems, Inc.) according to the manufacturer’s instructions. Briefly, slides were deparaffinized with a mild detergent: EZ-Prep (Ventana Medical Systems, Inc.) and a pretreatment was conducted with a combination of heat- and proteolytic-induced epitope retrieval steps. After pretreatment, the primary antibody was added and revealed with a DAB substrate chromogen (Dako Cytomation). Then,

the slides were counterstained and mounted. Images of tumor sections were acquired on a Leica microscope. Histological features and pathological diagnosis were made by a pathologist on the original hematoxylin and eosin (HE) stained slides.

RNA-sequencing

Clustering and sequencing (Novogene Experimental Department)

The clustering of the index-coded samples was performed on a cBot Cluster Generation System using TruSeq PE Cluster Kit v3-cBot-HS (Illumia) according to the manufacturer's instructions. After cluster generation, the library preparations were sequenced on an Illumina Hiseq platform and 125 bp/150 bp paired-end reads.

Data analysis

Quality control: Raw data (raw reads) of fastq format were firstly processed through in-house perl scripts. In this step, clean data (clean reads) were obtained by removing reads containing adapter, reads containing ploy-N and low quality reads from raw data. At the same time, Q20, Q30 and GC content the clean data were calculated. All the downstream analyses were based on the clean data with high quality.

Reads mapping to the reference genome

Reference genome and gene model annotation files were downloaded from genome website directly. Index of the reference genome was built using Hisat2 v2.0.5 and paired-end clean reads were aligned to the reference genome using Hisat2 v2.0.5.

We selected Hisat2 as the mapping tool for that Hisat2 can generate a database of splice junctions based on the gene model annotation file and thus a better mapping

result than other non-splice mapping tools.

Quantification of gene expression level

Feature Counts v1.5.0-p3 was used to count the reads numbers mapped to each gene. And then FPKM of each gene was calculated based on the length of the gene and reads count mapped to this gene. FPKM, expected number of Fragments Per Kilobase of transcript sequence per Millions base pairs sequenced, considers the effect of sequencing depth and gene length for the reads count at the same time, and is currently the most commonly used method for estimating gene expression levels.

Differential expression analysis

(For DESeq2 with biological replicates) Differential expression analysis of two conditions/groups (two biological replicates per condition) was performed using the DESeq2 R package (1.16.1). DESeq2 provide statistical routines for determining differential expression in digital gene expression data using a model based on the negative binomial distribution. The resulting P-values were adjusted using the Benjamini and Hochberg’s approach for controlling the false discovery rate. Genes with an adjusted P-value <0.05 found by DESeq2 were assigned as differentially expressed. (For edgeR without biological replicates) Prior to differential gene expression analysis, for each sequenced library, the read counts were adjusted by edgeR program package through one scaling normalized factor. Differential expression analysis of two conditions was performed using the edgeR R package (3.18.1). The P values were adjusted using the Benjamini & Hochberg method. Corrected P-value of 0.05 and absolute foldchange of 2 were set as the threshold for

significantly differential expression.

References:

1. Wang Z, Gerstein M, Snyder M. RNA-Seq: a revolutionary tool for transcriptomics [J]. Nature Reviews Genetics. 2009, 10(1): 57-63.
2. Parkhomchuk D, Borodina T, Amstislavskiy V, et al. Transcriptome analysis by strand-specific sequencing of complementary DNA [J]. Nucleic Acids Research. 2009, 37(18): e123-e123.
3. Daehwan Kim, Ben Langmead, Steven L Salzberg.HISAT: a fast spliced aligner with low memory requirements. Nature Methods. 2015, 12, 357–360.
4. Trapnell C, Pachter L, Salzberg S L. TopHat: discovering splice junctions with RNA-Seq [J].Bioinformatics. 2009, 25(9): 1105-1111.
5. Mortazavi A, Williams B A, McCue K, et al. Mapping and quantifying mammalian transcriptomes by RNA-Seq [J]. Nature Methods. 2008, 5(7): 621-628.
6. Liao Y1, Smyth GK, Shi W. featureCounts: an efficient general purpose program for assigning sequence reads to genomic features [J]. Bioinformatics. 2014, 30(7):923-30.
7. Garber M, Grabherr M G, Guttman M, et al. Computational methods for transcriptome annotation and quantification using RNA-seq [J]. Nature Methods. 2011, 8(6): 469-477.
8. Bray N, Pimentel H, Melsted P, et al. Near-optimal RNA-Seq quantification [J]. Nature Biotechnology. 2016, 34(5): 525-527.

9. Patro R, Mount S M, Kingsford C. Sailfish enables alignment-free isoform quantification from RNAseq reads using lightweight algorithms[J]. Nature Biotechnology. 2014, 32(5): 462-464.

10. Trapnell C, Roberts A, Goff L, et al. Differential gene and transcript expression analysis of RNA-seq experiments with TopHat and Cufflinks [J]. Nature Protocols. 2012, 7(3): 562-578.

11. Anders S, Huber W. Differential expression analysis for sequence count data [J]. Genome Biology. 2010, 11(10): R106.

12. Love M I, Huber W, Anders S. Moderated estimation of fold change and dispersion for RNA-seq data with DESeq2 [J]. Genome Biology. 2014, 15(12): 1-21.

13. Robinson M D, McCarthy D J, Smyth G K. edgeR: a Bioconductor package for differential expression analysis of digital gene expression data [J]. Bioinformatics. 2010, 26(1): 139-140.

14. Tarazona S, Garc á-Alcalde F, Dopazo J, et al. Differential expression in RNA-seq: a matter of depth [J]. Genome Research. 2011, 21(12): 2213-2223.

15. Young M D, Wakefield M J, Smyth G K, et al. Method Gene ontology analysis for RNA-seq: accounting for selection bias [J]. Genome Biology. 2010, 11: R14.

16. Kanehisa M, Goto S. KEGG: kyoto encyclopedia of genes and genomes [J]. Nucleic Acids Research. 2000, 28(1): 27-30.

17. Katz Y, Wang E T, Airoldi E M, et al. Analysis and design of RNA sequencing experiments for identifying isoform regulation [J]. Nature Methods. 2010, 7(12):

1009-1015.

18. McKenna A, Hanna M, Banks E, et al. The Genome Analysis Toolkit: a MapReduce framework for analyzing next-generation DNA sequencing data [J]. Genome Research. 2010, 20(9): 1297-1303.
19. Jia W, Qiu K, He M, et al. SOAPfuse: an algorithm for identifying fusion transcripts from paired-end RNA-Seq data [J]. Genome Biology. 2013, 14(2): R12.
20. Pastinen T. Genome-wide allele-specific analysis: insights into regulatory variation [J]. Nature Reviews Genetics. 2010, 11(8): 533-538.
21. Sun W. A statistical framework for eQTL mapping using RNA-seq data [J]. Biometrics. 2012, 68(1):1-111.
22. Giambartolomei C, Vukcevic D, Schadt E E, et al. Bayesian test for colocalisation between pairs of genetic association studies using summary statistics [J]. PLoS Genetics. 2014, 10(5): e1004383.
